# Evaluation of Analgesic Activity of* Papaver libanoticum* Extract in Mice: Involvement of Opioids Receptors

**DOI:** 10.1155/2017/8935085

**Published:** 2017-02-08

**Authors:** Mohamad Ali Hijazi, Ahmed El-Mallah, Maha Aboul-Ela, Abdalla Ellakany

**Affiliations:** ^1^Department of Pharmaceutical Sciences, Beirut Arab University, Beirut, Lebanon; ^2^Faculty of Pharmacy, Pharos University, Alexandria, Egypt; ^3^Department of Pharmaceutical Sciences, Faculty of Pharmacy, Beirut Arab University, Beirut, Lebanon

## Abstract

*Papaver libanoticum* is an endemic plant to Lebanese region (family Papaveraceae) that has not been investigated before. The present study aimed to explore the analgesic activity of dried ethanolic extract of* Papaver libanoticum* (PLE) using tail flick, hot plate, and acetic acid induced writhing models in mice. The involvement of opioid receptors in the analgesic mechanism was investigated using naloxone antagonism. Results demonstrated that PLE exhibited a potent dose dependent analgesic activity in all tested models for analgesia. The analgesic effect involved activation of opioid receptors in the central nervous system, where both spinal and supraspinal components might be involved. The time course for analgesia revealed maximum activity after three hours in both tail flick and hot plate methods, which was prolonged to 24 hours. Metabolites of PLE could be responsible for activation of opioid receptors. The EC50 of PLE was 79 and 50 mg/kg in tail flick and hot plate tests, respectively. The total coverage of analgesia by PLE was double that of morphine in both tests. In conclusion, PLE proved to have opioid agonistic activity with a novel feature of slow and prolonged effect. The present study could add a potential tool in the armaments of opioid drugs as a natural potent analgesic and for treatment of opioid withdrawal syndrome.

## 1. Introduction

Despite recent developments in pain therapies, the medical community still needs safe, effective, and potent analgesic drugs for the treatment of different painful conditions especially the chronic pain [1]. Thousands of patients with intense pain, such as that resulting from cancer or severe injury, must depend on current regimes (peripheral or centrally acting) like morphine, aspirin, and nonsteroidal anti-inflammatory drugs [2, 3]. Studies have shown that opiates cause physical dependency, tolerance, and addiction while NSAIDs usually cause gastrointestinal disorders [4, 5]. For that, the discovery of other alternatives to treat pain is crucial [6]. Herbal therapy could be an interesting option for the treatment of opioid dependence and withdrawal [7].

*Papaver libanoticum* is a member of genus* Papaver*, endemic to Lebanese region, family Papaveraceae [8]. It is a small herb with 5–30 cm stem, yellow-orange latex, and oblong leaves, covered with white silky hairs. It has orange-red petals, four stigmas, and black-blue capsule. It grows widely at higher altitudes as Cedars Mountain, Makmel Mountain, Sannine, and Qornet es-Saouda in Lebanon.* Papaver* species are very important medicinally as they are source for many pharmacologically active alkaloids [9].

*Papaver* alkaloids are well known for their anticarcinogenic, antioxidant, antimutagenic [10–12], antimicrobial, and anti-inflammatory [13] activities. Extracts from* Papaver* plant have been used for the treatment of diarrhea, cough, analgesia, and reduction of withdrawal signs of the opioid addiction [14]. Other studies also reported the ability of plant extract to inhibit morphine tolerance in mice [15]. The present study aimed to explore the analgesic potential of* Papaver libanoticum* extract and its effect on opioid receptor using different analgesic animal models.

## 2. Materials and Methods

### 2.1. Plant Material

Aerial parts of* Papaver libanoticum* were collected during the flowering period from Cedars Mountain of Lebanon (2000–2500 m above sea level) during August 2015. The plant was authenticated by Dr. George Tohme, Professor of taxonomy (National Council for Scientific Research (CNRS), Beirut, Lebanon). A voucher specimen (Pl-A-77-15) was deposited in the herbarium of the Faculty of Pharmacy, Beirut Arab University, Beirut, Lebanon. The plant was dried under shade at room temperature, and the dried aerial parts were ground into moderately coarse powder.

### 2.2. Preparation of Plant Extract

The air-dried and ground aerial parts of the plant (4 kg) were extracted successively with ethanol 86% (10 L × 4) at laboratory temperature. The residues were removed by filtration. The extract was concentrated in a rotary evaporator under reduced pressure at 40–50°C and lyophilized to get dried powder.

### 2.3. Phytochemical Analysis

#### 2.3.1. Determination of Total Alkaloid

50 g of the dried ethanolic extract residue was extracted with 10% sulphuric acid; then the combined filtered acidic extract was washed with diethyl ether and the ether washing discarded. The aqueous extract was rendered alkaline with concentrated ammonium hydroxide solution (PH = 9) and extracted with successive portions of chloroform and subsequently with chloroform : methanol (9 : 1) until no further alkaloid was detected. The combined organic extracts were washed with water, dried over anhydrous sodium sulphate, filtered, and concentrated to dryness under reduced pressure [16].

#### 2.3.2. Determination of Total Phenolic

The total phenolic content was measured using the Folin-Ciocalteu reagent (FCR) [17]. In brief, serial dilutions of PLE were prepared. A volume of 50 *µ*L of the extract was introduced into test tubes followed by 0.25 mL FCR. The solutions were kept at dark for 5 min. A 0.5 mL sodium carbonate (20% w/v) was added to the mixtures. Mixtures were vortexed and the volume was completed to 5 ml with distilled water. The preparations were kept in dark at room temperature for 30 min. The absorbance was measured at 765 nm using UV-vis (Jasco V-530) and compared to a gallic acid curve constructed using freshly prepared solutions. The results were expressed as mg gallic acid/g dried sample. Each assay was carried out in triplicate.

### 2.4. Acute Toxicity Test

The acute toxicity test was performed according to up-and-down method [18]. A group of mice (*n* = 6) were injected with PLE orally at a dose of 500, 1000, and 2000 mg/kg. The dose was increased as the animal survived at the smaller dose. The vehicle (DMSO + water) was used as a control and the animals were observed carefully during 24 h for any gross effect or mortality.

### 2.5. Animals

Pharmacological experiments were carried out using Swiss male albino mice weighing 20–25 g. Mice were raised in the animal house of the Faculty of Pharmacy of Beirut Arab University. The animals were randomly grouped in polyacrylic cages and maintained under standard animal housing conditions (temperature 25° ± 2°C) and relative humidity (40–70%) with dark-light cycles (12/12 h). The mice had free access to water ad libitum and standard laboratory chow. The mice were acclimatized to laboratory condition for 1 day before experimentation. Animals had no access to food during the whole day of experiment. Animal care and handling for the research were performed in accordance with the regulations and guidelines stipulated by the Institutional Animal Care and Use Guidelines (IACUG) at Beirut Arab University, Lebanon (IRB approval code: 2016A-0038-P-P-0142).

### 2.6. Animal Experimental Design

#### 2.6.1. Tail Flick and Hot Plate Test

Mice were divided into nine groups of 6 mice each. Group I served as negative control and received the vehicle (DMSO + water, 1 : 1), where groups II, III, IV, V, VI, VII, and VIII received PLE at doses 12.5, 25, 50, 75, 100, 150, and 200 mg/kg, respectively. Group IX was injected by morphine sulphate 5 mg/kg to act as positive control. All drugs were injected intraperitoneally (50 *µ*l), 30 min prior to the experiment. Pain was induced by placing on the hot plate meter and tail flick instrument. The latency time for responses was measured at different time intervals as described below.

#### 2.6.2. Test for Involvement of Opioid Receptors in the Nociceptive Effect

In order to test opioid receptors involvement, the animals were arranged in six groups of six mice each. Group I received the vehicle (control), group II received naloxone (4 mg/kg), and groups III and IV received PLE (100 mg/kg) and morphine (5 mg/kg), respectively, to serve as positive control. Groups V and VI were given naloxone 15 min prior to injection of PLE and morphine, respectively.

#### 2.6.3. Acetic Acid Induced Writhing

Animals were divided into six groups of 6 mice each. Group I received the vehicle (DMSO + water, 1 : 1) as a control. Groups II and III received the standard drugs morphine (5 mg/kg) and diclofenac (10 mg/kg), respectively. Groups IV received PLE 100 mg/kg. All injections were performed 30 min before injection of acetic acid. Group V received PLE 100 mg/kg 3 hours prior to acetic acid injection. Group VI received PLE 100 mg/kg 30 min prior to acetic acid injection but was treated with naloxone 4 mg/kg 15 min prior to administration of PLE.

### 2.7. Tail Flick Method

For the tail flick method pain was induced by giving radiant heat on the tail of the mice 5 cm away from the tip of the tail (using tail flick analgesic apparatus type 812, UGO BASILE®, Germany). Mice were held loosely in a towel during the test. Reaction time was recorded as the interval between exposing the tail to the light beam and the withdrawal of the tail. A cut-off time of 20 secs was imposed as a protection against tissue damage [19]. The test was done at 0, 0.5, 1, 1.5, 2, 3, 4, 6, and 8 hours. After 24 hours, the animals were tested again for any remaining activity. The change in latency time was calculated as *T* − *T*_0_ (where *T*_0_ is the latency at zero time).

### 2.8. Hot Plate Method

The hot plate test was used to measure response latency time according to the method described by Eddy and Leimbach [20]. Animals were placed on hot metal plate maintained at 55°C surrounded by a Plexiglas cylinder (height 26 cm, diameter 19 cm). The hot plate was provided by UGO BASILE HOT PLATE (Model 7280, Germany). Elapsed time between placement of the animal on the hot plate and the occurrence of the licking of the hind paws, shaking, or jump off from the surface was recorded as response latency in seconds. The specificity and sensitivity of the test were increased by measuring the reaction time of the first evoked behavior regardless of whether it is paw licking or jumping. The responses were measured at 0, 0.5, 1, 1.5, 2, 3, 4, 6, 8, and 24 hours. Only mice that showed initial nociceptive response within 30 seconds were selected and used for the study. The cut-off time for the hot plate latencies was set at 30 secs. The percentage protection against thermal pain stimulus was calculated according to the following formula [21]:

(1) Percentage  protection  against  thermal  stimulus=Test  mean  Ta−Control  mean TbControl mean Tb×100.

### 2.9. Acetic Acid Induced Writhing

The peripheral nociceptive activity of PLE was determined by the acetic acid abdominal constriction test [22, 23]. The writhes were induced by the intraperitoneal injection of 1% acetic acid (10 ml/kg). The numbers of writhes (muscular contractions) were counted 5 min after acetic acid injection over a period of 20 min. The number of writhes in each group was compared with the control and the percent reduction of writhes count was calculated as follows: (*N*_control_ − *N*_test_)/*N*_control_ × 100, where *N* is the mean number of writhes for each group.

### 2.10. Test for Involvement of Opioid Receptors in the Nociceptive Effect

The participation of the opioid system in the analgesic activity of PLE was examined by injecting naloxone hydrochloride (4 mg/kg, i.p., 50 *µ*l), a nonselective opioid receptor antagonist, 15 min prior to the administration of the test samples [24] as described above. Another dose of naloxone was given after 2 hours to maintain its concentration at a stable level in the animal. The hot plate, tail flick, and abdominal writhes tests were repeated as the same procedures described above. Latencies were sequentially measured at the same time intervals and cut-off time for the safety of animals.

### 2.11. Determination of EC50

The EC50 of PLE was determined by plotting the logarithmic concentrations of the extract versus the responses (change in latency time) in tail flick and hot plate tests to get the dose response curve (DRC).

### 2.12. Statistical Analysis

The results of the experiments were expressed as mean ± SEM (standard error of the mean). The mean values of control groups were compared with the mean value of treated groups using one-way ANOVA followed by post hoc analysis. Results were considered statistically significant when *P* values were <0.05 [25]. MegaStat and GraphPad Prism (version 4) were used for statistical analysis. The total coverage of analgesia was determined by calculating the Area under the Curve (AUC) using the trapezoidal method. Both EC50 and AUC were calculated using GraphPad Prism software.

## 3. Results

### 3.1. Phytochemical Analysis

The results of the phytochemical analysis revealed a total alkaloidal content of 2.45 g (from 50 g PLE); thus the yield was 4.9%. The total phenolic content is measured using a calibration curve obtained with known concentrations of gallic acid standard, which was determined as 2.6 ± 0.01 mg gallic acid/g dried extract.

### 3.2. Tail Flick Test

**Figure 1:**
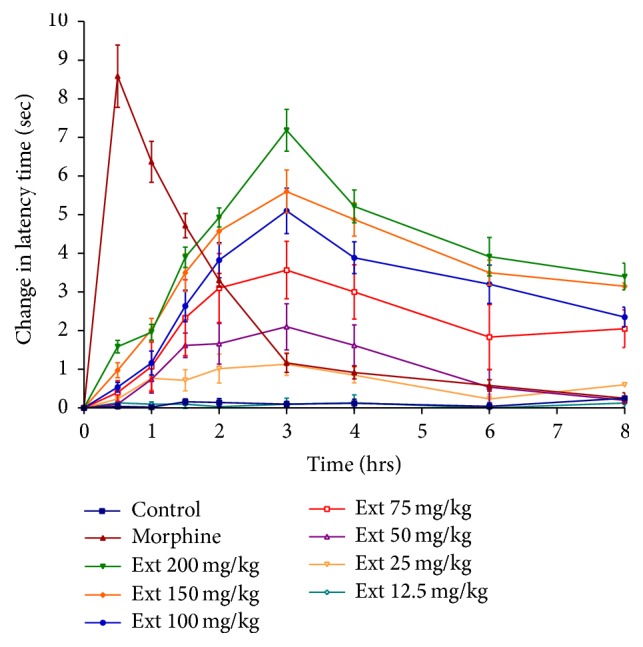
Effect of different doses of PLE on change in latency time using tail flick test in mice.

**Figure 2:**
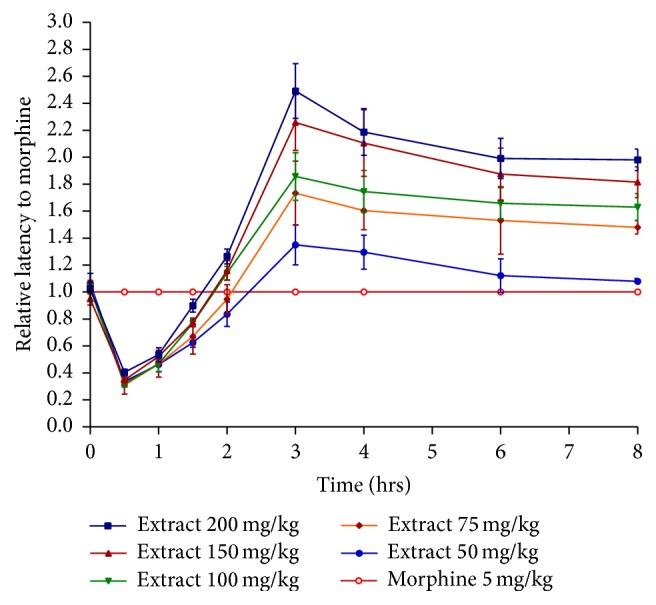
Relative activity of PLE with respect to morphine in tail flick method.

The results of the analgesic activity of dried ethanolic extract of* Papaver libanoticum* are shown in Table 1. Control group of mice (injected by vehicle) did not show any significant difference in the reaction time on tail flick throughout the whole observation time. PLE (50, 75, 100, 150, and 200 mg/kg) revealed a significant and dose dependent increase in the latency time when compared to the control group. The maximum reaction time for morphine (standard) was 11.31 secs reached at 30 min, which returns to normal after four hrs (3.77 secs). On the other hand, the maximum activity of PLE appeared after 3 hours (5.2, 6.5, 7.4, 8.2, and 10 secs for 50, 75, 100, 150, and 200 mg/kg doses, resp.) as can be seen in Table 1. These increases in latencies (for PLE) remain significant after 8 hours and even after 24 hours (Figure 1). The relative activity of PLE with respect to morphine in tail flick test was shown in Figure 2. The figure illustrated that after 3 hours all doses (50, 75, 100, 150, and 200 mg/kg) of PLE were more effective than morphine with relative activity of 1.4, 1.47, 1.86, 2.26, and 2.49, respectively. The difference in the activity between PLE and morphine after three hrs (until eight hrs) were statistically significant. Morphine was only more potent between zero and two hours. On the other hand, PLE reached its peak activity after 3 hours indicating a slow onset of action, but its analgesic activity extended significantly even after 8 hours. By comparing coverage of analgesia by time, the AUC of PLE 200 mg/kg (33.58) was double that of morphine (16.85) with peak response (change in latency) of 7.18 and 8.5 secs, respectively. All other doses (75, 100, and 150 mg/kg) showed also greater coverage of analgesia than morphine.

### 3.3. Hot Plate Test

**Figure 3:**
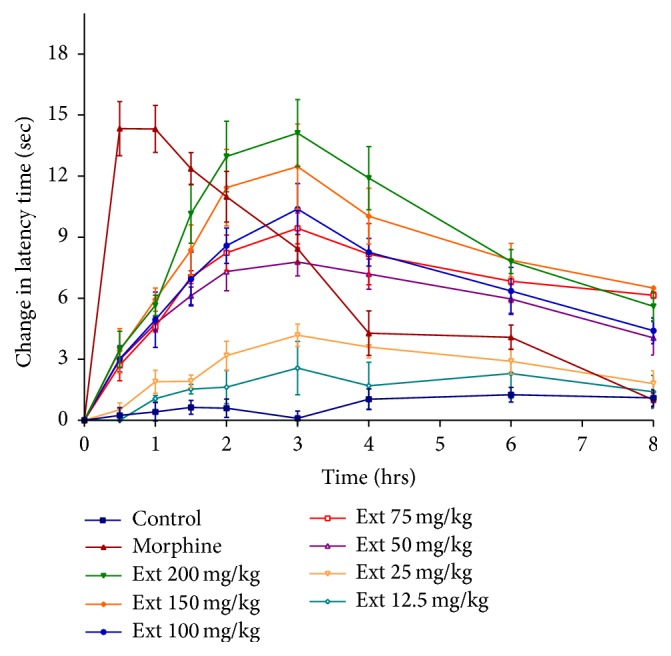
Effect of different doses of PLE on change in latency time using hot plate test in mice.

**Figure 4:**
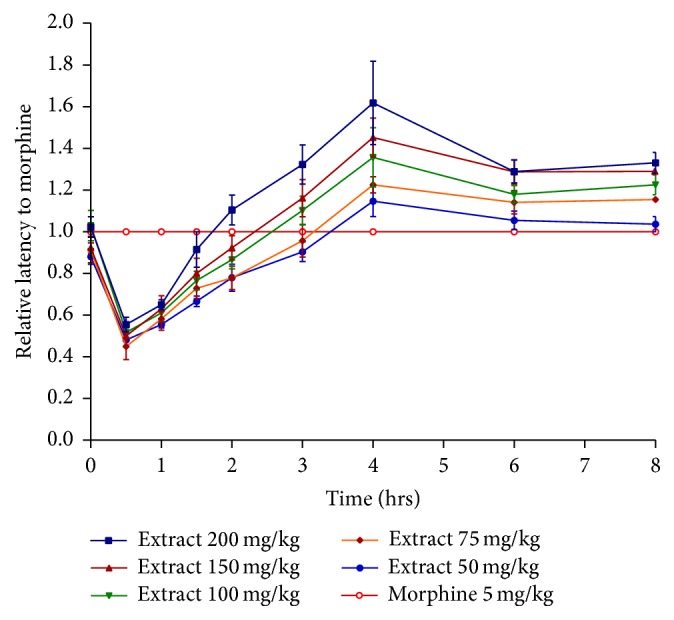
Relative activity of PLE with respect to morphine in hot plate method.

The results of the analgesic activity of PLE using hot plate method are presented in Table 2. There was no significant difference on the thermal stimulus in mice treated with the vehicle (negative control) throughout the whole time of the experiment. Morphine administration significantly increased response time of the animal to reach 24 secs (after 0.5 hrs–1 hr). Its analgesic effect decreased with time but remained significant even after 6 hours (13.8 secs). All doses except 12.5 mg/kg of PLE showed significant increase in the latency time of mice when compared to control group. As can be seen in Table 2, the maximum activities were obtained after 3 hours with 15.7, 16.8, 18, 18.8, 21.3, and 24 secs for 50, 75, 100, 150, and 200 mg/kg PLE. The increase in latency time induced by the plant extract was maintained for 8 hours and remained significant after 24 hrs for 100, 150, and 200 mg/kg doses (Figure 3).  Figure 4 demonstrated the relative activity of PLE with respect to the standard drug (morphine) in the hot plate method. Similar to tail flick results, all doses of PLE were more effective than morphine after 3.5 hrs. The maximum activity of PLE was achieved at 4 hours, where its relative activity to morphine reached 1.15, 1.1, 1.36, 1.4, and 1.62 for 50, 75, 100, 150, and 200 mg/kg, respectively. The analgesic effect of the extract was very close to morphine with relative activities greater than 1 for all doses after 3 hours. The peak in latency time response of morphine (after 30 min) was 14.33, while that of PLE was 14.12 secs (200 mg/kg) after 3 hours. The time of analgesic coverage calculated as AUC for all doses of PLE (75, 100, 150, and 200 mg/kg) was 53.9, 54.2, 67.7, and 72.9 respectively, being greater than that of morphine 52.83, as can be seen in Table 3.

### 3.4. Naloxone Antagonism

**Figure 5:**
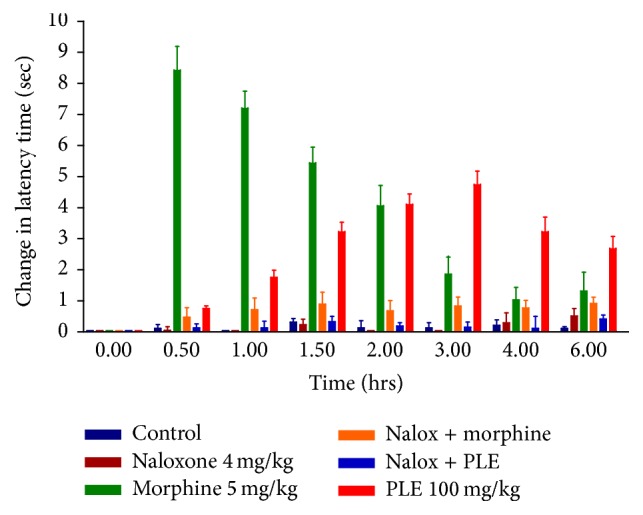
Effect of naloxone on latency time change in tail flick test in mice.

**Figure 6:**
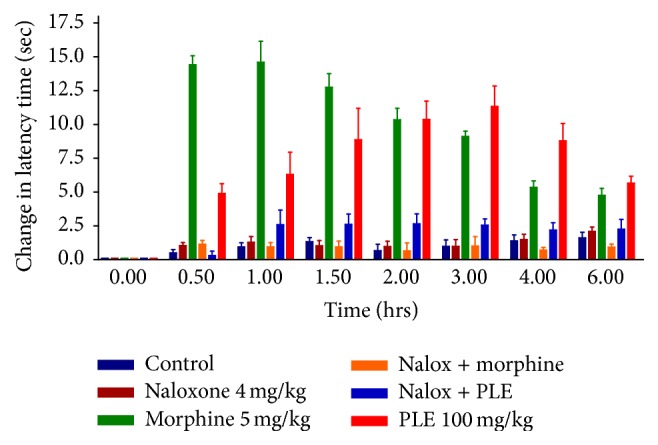
Effect of naloxone on latency time change in hot plate test in mice.

In order to assess the involvement of the opioid receptor in the pain relieving effect of PLE, naloxone was injected, 15 min prior to the administration of test samples. The group that received naloxone alone did not show any increase in latency time (in tail flick and hot plate tests) as can be seen in Tables 4 and 5. The analgesic effect of morphine was completely antagonized in both hot plate and tail flick methods indicating the validity of the experiments. Antinociceptive effect induced by PLE (100 mg/kg) was completely inhibited in animals treated with naloxone in both hot plate and tail flick tests (Figures 5 and 6).

### 3.5. Total Analgesia Coverage (AUC)

The total coverage time of analgesia was determined by calculating the Area under the Curve (AUC) of response versus time in both tail flick and hot plate tests. Results are summarized in Table 3. In both tests, morphine reached its peak response (8.5 or 14.33 secs) after 0.5 hrs, while PLE reached its maximum response after 3 hours for all doses. In tail flick method, the total analgesia coverage of morphine (16.85) was close to that of PLE at 75 mg/kg (18 secs). On the other hand, higher doses (100, 150, and 200 mg/kg) of PLE revealed double the AUC of morphine with 23.3, 29.6, and 33.8, respectively. The AUC using hot plate test was 52.8 for morphine and 53.9, 54.2, 67.7, and 72.9 secs for PLE at 75, 100, 150, and 200 mg/kg, respectively.

### 3.6. EC50 of PLE

**Figure 7:**
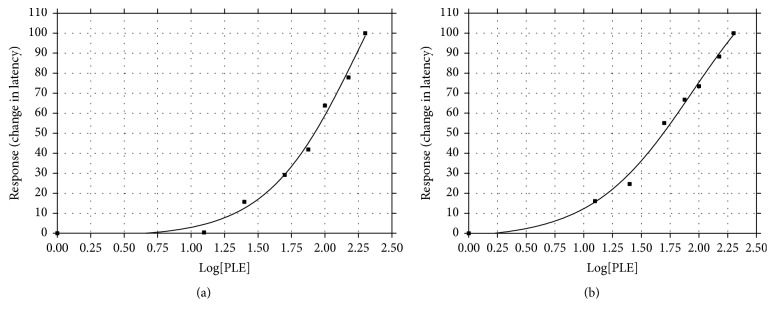
DRC of PLE in tail flick (a) and hot plate (b) tests in mice.

The resulting dose response curves of PLE in tail flick and hot plate tests were shown in Figure 7. The EC50 of a dose response curve represents the concentration of a compound where 50% of its maximal effect is observed. The EC50 of PLE was calculated as 79 mg/kg and 50 mg/kg in tail flick and hot plate tests, respectively.

### 3.7. Acetic Acid Induced Writhing

**Figure 8:**
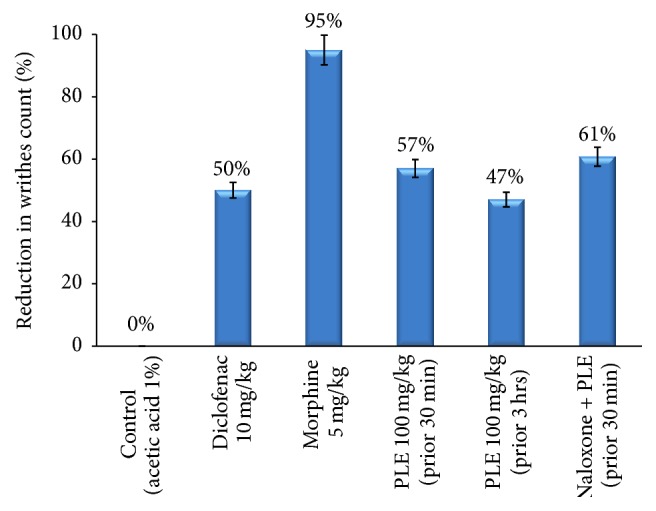
Percentage of inhibition of abdominal contractions in acetic acid induced writhing in mice.

Regarding the results of PLE in the acetic acid induced writhing test, a highly significant reduction in the writhes count was recorded as compared to control group (Table 6 and Figure 8). Morphine showed the highest protection against the acetic acid induced writhing (95%), while diclofenac (standard) showed 50% reduction in the writhes count. PLE given 30 min prior to acetic acid injection revealed greater protection than that given before 3 hours (57% and 47%, resp.). No significant differences were obtained between PLE and diclofenac drug in the reduction of writhes count.

## 4. Discussion

The ED50 of PLE was found to be more than 2000 mg/kg upon being given orally in mice. The extract showed no gross morbidity, except some sedation that was resolved after about six hours. No mortality was recorded during 24 h even for a single animal. Analgesics are drugs that act on central or peripheral nervous system to relieve pain selectively without altering consciousness [26]. Centrally acting analgesics act by increasing the threshold for pain and altering the physiological response to pain. However, peripherally acting drugs act by inhibiting the generation of pain impulses at the chemoreceptor level [27]. The analgesic activity of PLE was studied by the tail flick, hot plate, and acetic acid induced writhing tests, which are standard pharmacological models for the assessment of analgesia by natural products [28]. Both tail flick and hot plate methods are used generally for centrally acting analgesic [29], while peripherally acting drugs are ineffective in these tests but sensitive to acetic acid induced writhing test [30].

In tail flick test, PLE, in a concentration dependent manner, exhibited significant antinociceptive activity by increasing the latency time of responses in mice in all tested doses except for 12.5 and 25 mg/kg, as can be seen in Table 1. In comparison to control, morphine (standard drug) produced the highest analgesic activity among all tested samples, as shown in Figure 1. Its peak activity was reached after half an hour. Morphine is considered as a potent analgesic drug that activates opioid receptor (*µ*, *δ*, and *κ*) [31]. The activation of these receptors has been associated with spinal, supraspinal, and peripheral analgesia [32]. Despite its rapid onset of action, morphine is a short acting drug, and its analgesic effect disappeared after 4 hours. This profile is in concordance with the known pharmacodynamics of morphine [33].

Again, data from the hot plate test emphasized the analgesic activity of PLE. PLE showed a dose and time dependent strong analgesic activity as compared to control group.

The slow onset and long duration of analgesic activity of the extract suggested an active metabolite that could be more effective than the prodrug in the extract and/or binding to plasma protein. The centrally mediated analgesia integrated response is affected mostly by opioids receptors [34]. Taken together, and since tail flick is a spinal reflex [35] and hot plate is mostly supraspinally integrated response, PLE could exhibit a significant and potential analgesic activity via activation of opioid receptors in the central nervous system. This hypothesis was verified by naloxone antagonism of the antinociceptive effect of PLE that was complete in both tail flick and hot plate models (Tables 4 and 5). Naloxone at the given concentration also diminished morphine-induced latency time in both experimental models.

Naloxone is a nonselective opioid receptor antagonist [36], with a short acting activity (half-life 64–78 min) [33], for that a second dose was administered to mice after 2 hours to maintain its plasma level. In both tail flick and hot plate tests, the group that received naloxone alone did not show any increase in latency time eliminating any agonistic activity of naloxone.

The differences in potencies obtained in the tail flick and hot plate tests could show the complexity in the type and mechanism of pain [37].

The acetic acid induced abdominal constriction test is used frequently for peripherally acting drugs. The pain induction occurs by liberating endogenous substances as well as some other pain mediators such as arachidonic acid metabolites via cyclooxygenases, such as prostaglandins [38]. It was observed that PLE significantly (*P* < 0.01) reduced the abdominal contractions induced by acetic acid even after 30 min of administration, in contrast to tail flick or hot plate tests (no activity was obtained after 30 min). Moreover, the activity of the extract administered 30 min prior to acetic acid injection was not completely antagonized by naloxone injection (61% reduction in writhes count), indicating that the antinociceptive effect was not totally mediated through opioid receptors. Therefore, it could be suggested that PLE might contain pharmacologically active constituents (other than those involved in the central analgesia) that can block the release or the effect of endogenous substances responsible for the excitation of nerve endings. The efficacy of peripheral analgesia of PLE (100 mg/kg) was comparable to diclofenac (10 mg/kg) where no significant difference was obtained in the protection against acetic acid induced writhing. Morphine revealed a complete inhibition of writhes in mice with 95% reduction in the writhes count. From mechanistic point of view, narcotic analgesics inhibit both peripherally and centrally mediated pain [39]. Thus, PLE contains two types of components responsible for the antinociceptive mechanisms. Some components in the PLE act centrally via activation of opioid receptors, after being metabolized (due to their effects after 2-3 hrs) where others act peripherally by inhibiting endogenous pain substances without any delay in onset.

*Papaver* species other than* Papaver somniferum* do not contain morphine or any of its substituted compounds [40]. Thus, the central analgesic activity of PLE may be attributed to the presence of alkaloids probably belonging to different classes (isoquinolines, aporphine, protopine, etc.) and polyphenolic compounds. It was reported that* Papaver rhoeas* extract exerted mild opioid activity [41] and it could reduce the withdrawal signs of morphine [42]. Isoquinoline alkaloids isolated from plant of family Papaveraceae were also shown to reduce withdrawal syndrome [43] especially berberine [44]. In previous work, we isolated five isoquinoline alkaloids (including berberine) from Lebanese* Papaver rhoeas* (under publication).

The finding that PLE is acting as an opioid agonist with its slow onset and prolonged effect suggested that it could be a potential tool for treatment of opioid withdrawal syndrome like methadone. Methadone is a commonly used drug in the treatment of narcotic abuse [33]. One of the most important pharmacological characteristics that support its use as a replacement therapy in the long-term treatment of opiate addiction is its long duration of action that makes a single daily administration possible [45]. The average time of methadone to reach the *T*_max_ is 2.5–4.4 hrs [46, 47].

## 5. Conclusion

The present study demonstrates that* Papaver libanoticum* extract acts as a potent analgesic agent. The analgesic activity may be due to its ability to activate opioid receptors in the central nervous system. It may also inhibit endogenous pain substances, which are involved in the peripheral analgesia. The analgesic activity of PLE may be due to the presence of alkaloids and other polyphenolic compounds. The agonistic activity of PLE with the characteristic profile of slow onset and prolonged duration could suggest PLE as a potential medication for treatment of opioid abuse and withdrawal symptoms.

## Figures and Tables

**Table 1:** Analgesic effect of different doses of PLE by tail flick method in mice.

Treatment	Reaction time (sec, mean ± SEM)									
	0 hrs	0.5 hrs	1 hr	1.5 hrs	2 hrs	3 hrs	4 hrs	6 hrs	8 hrs	24 hrs
Control (vehicle)	2.78 ± 0.13	2.82 ± 0.18	2.8 ± 0.14	2.86 ± 0.23	2.92 ± 0.2	2.88 ± 0.04	2.90 ± 0.28	2.82 ± 0.23	3 ± 0.28	2.9 ± 0.25
PLE 12.5 mg/kg	2.76 ± 0.05	2.9 ± 0.1	2.87 ± 0.15	2.8 ± 0.26	2.8 ± 0.1	2.8 ± 0.36	2.9 ± 0.36	2.73 ± 0.15	2.83 ± 0.25	2.8 ± 0.2
PLE 25 mg/kg	2.57 ± 0.24	2.8 ± 0.23	3.33 ± 0.72	3.28 ± 0.5	3.58 ± 0.78	3.7 ± 0.64	3.42 ± 0.54	2.8 ± 0.27	2.95 ± 0.21	2.85 ± 0.07
PLE 50 mg/kg	3.14 ± 0.11	3.24 ± 0.11	3.88^*∗*^ ± 0.8	4.76^*∗∗*^ ± 0.73	4.8^*∗*^ ± 1.15	5.24^*∗∗*^ ± 1.3	4.76^*∗*^ ± 1.13	3.68^*∗*^ ± 0.97	3.4 ± 0.14	2.85 ± 0.07
PLE 75 mg/kg	2.93 ± 0.42	3 ± 0.6	4^*∗*^ ± 0.7	5.27^*∗∗*^ ± 1.32	6.03^*∗∗*^ ± 1.19	6.5^*∗∗*^ ± 0.95	5.93^*∗∗*^ ± 0.95	4.77^*∗*^ ± 1.16	3.95^*∗*^ ± 0.49	3.05 ± 0.21
PLE 100 mg/kg	2.87 ± 0.17	3.51 ± 0.24	4.20^*∗*^ ± 0.76	5.84^*∗∗*^ ± 0.66	7.09^*∗∗*^ ± 0.8	7.46^*∗∗*^ ± 0.82	6.44^*∗∗*^ ± 0.83	5.63^*∗∗*^ ± 0.67	5.15^*∗∗*^ ± 0.78	4^*∗*^ ± 0.14
PLE 150 mg/kg	2.65 ± 0.13	3.63 ± 0.43	4.68^*∗∗*^ ± 0.57	6.15^*∗∗*^ ± 0.9	7.23^*∗∗*^ ± 0.61	8.25^*∗∗*^ ± 0.99	7.53^*∗∗*^ ± 0.75	6.15^*∗∗*^ ± 0.6	5.7^*∗∗*^ ± 0.1	4.67^*∗*^ ± 0.25
PLE 200 mg/kg	2.89 ± 0.17	4.47^*∗*^ ± 0.44	4.84^*∗∗*^ ± 0.47	6.79^*∗∗*^ ± 0.63	7.81^*∗∗*^ ± 0.55	10.07^*∗∗*^ ± 1.32	8.1^*∗∗*^ ± 1.07	6.8^*∗∗*^ ± 1.26	6.25^*∗∗*^ ± 0.78	5.05^*∗*^ ± 0.21
Morphine (5 mg/kg)	2.81 ± 0.18	11.31^*∗∗*^ ± 1.92	9.30^*∗∗*^ ± 1.36	7.61^*∗∗*^ ± 0.73	6.23^*∗∗*^ ± 0.47	4.16^*∗*^ ± 0.79	3.77 ± 0.46	3.44 ± 0.39	3.15 ± 0.21	3 ± 0.14

^*∗*^ *P* < 0.05; ^*∗∗*^*P* < 0.001 (*n* = 6).

**Table 2:** Analgesic effect of different doses of PLE by hot plate method in mice.

Treatment	Reaction time (sec, mean ± SEM)									
	0 hrs	0.5 hrs	1 hr	1.5 hrs	2 hrs	3 hrs	4 hrs	6 hrs	8 hrs	24 hrs
Control	9.4 ± 0.54	9.64 ± 0.91	9.82 ± 0.68	10.04 ± 0.42	10 ± 0.7	9.5 ± 0.65	10.44 ± 0.93	10.66 ± 0.65	10.2 ± 0.28	9.15 ± 0.35
PLE 12.5 mg/kg	8.6 ± 0.75	8.23 ± 0.65	9.67 ± 1	10.13 ± 0.64	9.87 ± 1.53	10.87 ± 2.14	10.1 ± 1.51	10.8 ± 1.91	10.77 ± 1.96	9.57 ± 1.1
PLE 25 mg/kg	9.32 ± 0.86	9.84 ± 1.04	11.22 ± 1.36	11.24 ± 1.2	12.50 ± 1.12	13.5^*∗*^ ± 0.56	12.92^*∗*^ ± 0.77	12.22^*∗*^ ± 0.62	11.5 ± 0.42	10.55 ± 0.21
PLE 50 mg/kg	8.53 ± 0.77	11.52 ± 1.64	13.28^*∗*^ ± 1.02	14.65^*∗∗*^ ± 0.82	15.85^*∗∗*^ ± 2.03	16.32^*∗∗*^ ± 1.37	15.72^*∗∗*^ ± 1.45	14.5^*∗∗*^ ± 1.26	12.55 ± 1.06	10.55 ± 0.64
PLE 75 mg/kg	8.63 ± 0.6	11.33 ± 1.72	13.23^*∗*^ ± 0.67	15.67^*∗∗*^ ± 0.31	16.87^*∗∗*^ ± 2.1	18.07^*∗∗*^ ± 1.92	16.8^*∗∗*^ ± 2	15.47^*∗∗*^ ± 1.21	13.83^*∗*^ ± 1.16	11 ± 0.2
PLE 100 mg/kg	9.82 ± 0.95	12.84^*∗*^ ± 1.79	14.76^*∗∗*^ ± 2.38	16.76^*∗∗*^ ± 2.09	18.4^*∗∗*^ ± 1.29	20.2^*∗∗*^ ± 2.31	18.08^*∗∗*^ ± 1.04	16.18^*∗∗*^ ± 1.87	14.7^*∗*^ ± 0.71	12.8^*∗*^ ± 0.57
PLE 150 mg/kg	8.83 ± 1	12.27 ± 1.92	14.77^*∗∗*^ ± 1.44	17.2^*∗∗*^ ± 2.35	20.27^*∗∗*^ ± 2.77	21.3^*∗∗*^ ± 3.11	18.87^*∗∗*^ ± 1.86	16.7^*∗∗*^ ± 0.44	15.83^*∗*^ ± 0.35	13.33^*∗*^ ± 0.61
PLE 200 mg/kg	9.88 ± 0.75	13.37^*∗∗*^ ± 2.46	15.53^*∗∗*^ ± 1.04	20.03^*∗∗*^ ± 3.59	22.85^*∗∗*^ ± 4.4	24^*∗∗*^ ± 4.41	21.78^*∗∗*^ ± 3.9	17.68^*∗∗*^ ± 1.01	15.9^*∗*^ ± 0.99	14.15^*∗*^ ± 1.06
Morphine (5 mg/kg)	9.72 ± 0.68	24.05^*∗∗*^ ± 2.67	24.03^*∗∗*^ ± 2.4	22.08^*∗∗*^ ± 1.44	20.7^*∗∗*^ ± 2.69	18.15^*∗∗*^ ± 1.14	14^*∗*^ ± 2.39	13.8^*∗*^ ± 1.08	10.8 ± 0.28	9.4 ± 0.28

^*∗*^ *P* < 0.05; ^*∗∗*^*P* < 0.001 (*n* = 6).

**Table 3:** Comparison of AUC of different doses of PLE and morphine.

Treatment	Tail flick method			Hot plate method		
	AUC	Response peak (sec)	Time (hrs)	AUC	Response peak (sec)	Time (hrs)
Morphine (5 mg/kg)	16.85	8.5	0.5	52.83	14.33	0.5
PLE 75 mg/kg	18.04	3.57	3	53.93	9.44	3
PLE 100 mg/kg	23.38	4.59	3	54.26	10.38	3
PLE 150 mg/kg	29.66	5.6	3	67.73	12.47	3
PLE 200 mg/kg	33.58	7.18	3	72.96	14.12	3

**Table 4:** Effect of naloxone on analgesic activity of PLE using tail flick method in mice.

Treatment	Reaction time (sec, mean ± SEM)							
	0 hrs	0.5 hrs	1 hr	1.5 hrs	2 hrs	3 hrs	4 hrs	6 hrs
Control (vehicle)	2.64	2.72	2.54	2.92	2.74	2.74	2.82	2.72
Naloxone (4 mg/kg)	2.68 ± 0.5	2.70 ± 0.3	2.62 ± 0.29	2.88 ± 0.19	2.62 ± 0.18	2.54 ± 0.34	2.94 ± 0.27	3.16 ± 0.29
Morphine 5 mg/kg	2.64 ± 0.24	11.04^*∗∗*^ ± 2.14	9.82^*∗∗*^ ± 1.3	8.06^*∗∗*^ ± 0.99	6.68^*∗∗*^ ± 1.48	4.46^*∗∗*^ ± 0.99	3.64 ± 0.71	3.92 ± 1.17
PLE 100 mg/kg	2.87 ± 0.17	3.51 ± 0.24	4.20^*∗*^ ± 0.76	5.84^*∗∗*^ ± 0.66	7.09^*∗∗*^ ± 0.8	7.46^*∗∗*^ ± 0.82	6.09^*∗∗*^ ± 0.94	5.53^*∗∗*^ ± 0.78
Naloxone + morphine	2.44 ± 0.27	2.88 ± 0.63	3.12 ± 0.67	3.30 ± 0.89	3.08 ± 0.64	3.24 ± 0.43	3.18 ± 0.43	3.32 ± 0.43
Naloxone + PLE	2.55 ± 0.23	2.63 ± 0.21	2.67 ± 0.33	2.85 ± 0.26	2.70 ± 0.35	2.72 ± 0.35	2.65 ± 0.59	2.93 ± 0.28

^*∗*^ *P* < 0.05; ^*∗∗*^*P* < 0.001 (*n* = 6).

**Table 5:** Effect of naloxone on analgesic activity of PLE using hot plate method in mice.

Treatment	Reaction time (sec, mean ± SEM)							
	0 hrs	0.5 hrs	1 hr	1.5 hrs	2 hrs	3 hrs	4 hrs	6 hrs
Control (vehicle)	8.7	9.12	9.38	9.94	9.14	9.46	9.92	10.22
Naloxone (4 mg/kg)	8.56 ± 0.92	9.52 ± 0.98	9.76 ± 1.16	9.50 ± 0.86	9.44 ± 0.71	9.46 ± 0.63	9.96 ± 0.74	10.56 ± 0.36
Morphine 5 mg/kg	9 ± 0.77	23.33^*∗∗*^ ± 1.89	23.5^*∗∗*^ ± 4.45	21.67^*∗∗*^ ± 2.95	19.27^*∗∗*^ ± 2.28	18.02^*∗∗*^ ± 1.23	14.25^*∗∗*^ ± 0.93	13.67^*∗∗*^ ± 1.32
PLE 100 mg/kg	9.68 ± 0.92	14.15^*∗∗*^ ± 1.09	15.30^*∗∗*^ ± 2.99	17.62^*∗∗*^ ± 4.43	19.33^*∗∗*^ ± 2.56	20.7^*∗∗*^ ± 2.58	17.88^*∗∗*^ ± 2.34	14.57^*∗∗*^ ± 1.5
Naloxone + morphine	7.86 ± 0.74	8.92 ± 0.53	8.72 ± 0.25	8.72 ± 1.28	8.42 ± 1.43	8.78 ± 1.57	8.48 ± 0.85	8.70 ± 0.93
Naloxone + PLE	7.63 ± 0.68	7.77 ± 0.74	9.57 ± 2.08	9.52 ± 1.59	9.68 ± 1.38	9.62 ± 0.98	9.22 ± 1.02	9.38 ± 1.36

^*∗∗*^ *P* < 0.001 (*n* = 6).

**Table 6:** Analgesic activity of PLE by acetic acid induced writhing in mice.

Group	Number of writhes (mean ± SEM)	Reduction in writhes count (%)
Control (acetic acid 1%)	59.6 ± 6.5	0%
Diclofenac 10 mg/kg	29.6^*∗∗*^ ± 3.9	50%
Morphine 5 mg/kg	3.2^*∗∗*^ ± 0.8	95%
PLE 100 mg/kg (prior 30 min)	25.4^*∗∗*^ ± 3	57%
PLE 100 mg/kg (prior 3 hrs)	31.6^*∗∗*^ ± 3	47%
Naloxone + PLE (prior 30 min)	23.4^*∗∗*^ ± 2.4	61%

^*∗∗*^ *P* < 0.001 (*n* = 6).
